# Inverted epiretinal proliferation flap with internal limiting membrane preservation for myopic macular hole retinal detachment: a case report

**DOI:** 10.3389/fopht.2026.1960225

**Published:** 2026-10-26

**Authors:** Zhi Zhong, Shuxin Cai, Haibo Li

**Affiliations:** 1 Xiamen Eye Center and Eye Institute of Xiamen University, School of Medicine, Xiamen, Fujian, China; 2 Zherong County Hospital, Ningde, Fujian, China

**Keywords:** epiretinal proliferation, high myopia, internal limiting membrane, macular hole retinal detachment, pars plana vitrectomy

## Abstract

In this case report, we describe a 52-year-old woman with high myopia presented with a macular hole retinal detachment for 5 months. Fundus examination showed a full-thickness macular hole with localized retinal detachment, accompanied by both superior and inferior posterior scleral staphylomas. Spectral-domain optical coherence tomography confirmed a large full-thickness macular hole with associated epiretinal proliferation. She underwent 27-gauge vitrectomy using an inverted epiretinal proliferation flap technique without internal limiting membrane peeling, followed by gas tamponade. Postoperatively, the retina was reattached and the macular hole was closed. Best-corrected visual acuity improved from hand motions to 20/200. At the 12-month final follow-up, BCVA remained stable at 20/200, with no evidence of hole reopening or recurrent retinal detachment. This case demonstrates that inverted epiretinal proliferation (ERP) flap with internal limiting membrane (ILM) preservation may represent a feasible tissue-preserving reconstructive strategy for selected patients with myopic macular hole retinal detachment (MHRD) when sufficient ERP is present.

## Introduction

Macular hole retinal detachment (MHRD) is a vision-threatening complication of high myopia characterized by retinal thinning, posterior scleral staphyloma, and complex vitreoretinal traction (1). Although pars plana vitrectomy with internal limiting membrane (ILM) peeling remains the standard treatment, anatomical closure and visual recovery remain suboptimal in eyes with advanced myopic degeneration (2).

The inverted ILM flap technique has significantly improved surgical outcomes and has become the preferred approach for large or complex myopic macular holes (3). More recently, several tissue-based reconstructive strategies, including free ILM flaps (4), lens capsule transplantation (5), human amniotic membrane grafting (6), and autologous retinal transplantation (7), have been introduced to provide biological support when conventional ILM flap surgery is not feasible. However, these techniques may be limited by insufficient donor tissue, additional surgical manipulation, or the need for exogenous materials.

Epiretinal proliferation (ERP) has emerged as a potential autologous scaffold for lamellar macular hole repair (8), but its application in highly myopic MHRD reconstruction remains largely unexplored. Herein, we describe a patient with high myopia and MHRD associated with multiple posterior scleral staphylomas who was successfully treated using an inverted ERP flap while preserving the ILM, highlighting a potential tissue-preserving alternative for carefully selected cases.

## Case presentation

A 52-year-old woman with high myopia presented with progressive blurred vision in her right eye for 5 months. She had undergone phacoemulsification with intraocular lens implantation in both eyes 10 years prior. She had no history of ocular trauma, glaucoma, diabetic retinopathy, or previous vitreoretinal surgery. Her systemic medical history was unremarkable. The patient’s pre-symptom visual acuity was not documented as she had not undergone regular ophthalmic follow-up. At presentation, best-corrected visual acuity (BCVA) was hand motions in the right eye. The BCVA was 20/160 in the left eye.

Anterior segment examination was unremarkable. Her axial length was 33.89 mm in the right eye, confirming high myopia. Fundus examination revealed a large full-thickness macular hole associated with localized retinal detachment involving the posterior pole. Distinct superior and inferior posterior scleral staphylomas were also observed, resulting in an irregular posterior pole configuration (**Figure 1A**). Spectral-domain optical coherence tomography (SD-OCT) demonstrated a full-thickness macular hole with a minimum diameter of 687 μm, accompanied by extensive ERP along the hole margin in the right eye (**Figure 1C**) and a lamellar macular hole with epiretinal membrane in the left eye. Localized retinal detachment corresponded to the superior posterior scleral staphyloma. After informed consent was obtained, the patient underwent 27-gauge pars plana vitrectomy using an inverted ERP flap technique with preservation of the ILM (**Supplementary Video 1**). Following core vitrectomy and complete posterior hyaloid separation, residual cortical vitreous was carefully removed. To confirm ILM preservation and facilitate differentiation of ERP from the ILM, indocyanine green was applied during surgery. The ERP surrounding the macular hole was meticulously dissected to create a continuous pedicled flap while preserving its retinal attachment and the underlying ILM. The ERP flap was then inverted to cover the macular hole using an ophthalmic viscosurgical device–assisted press injection technique (9). Fluid–air exchange was subsequently performed, followed by 15% perfluoropropane (C_3_F_8_) gas tamponade.

**Figure 1:**
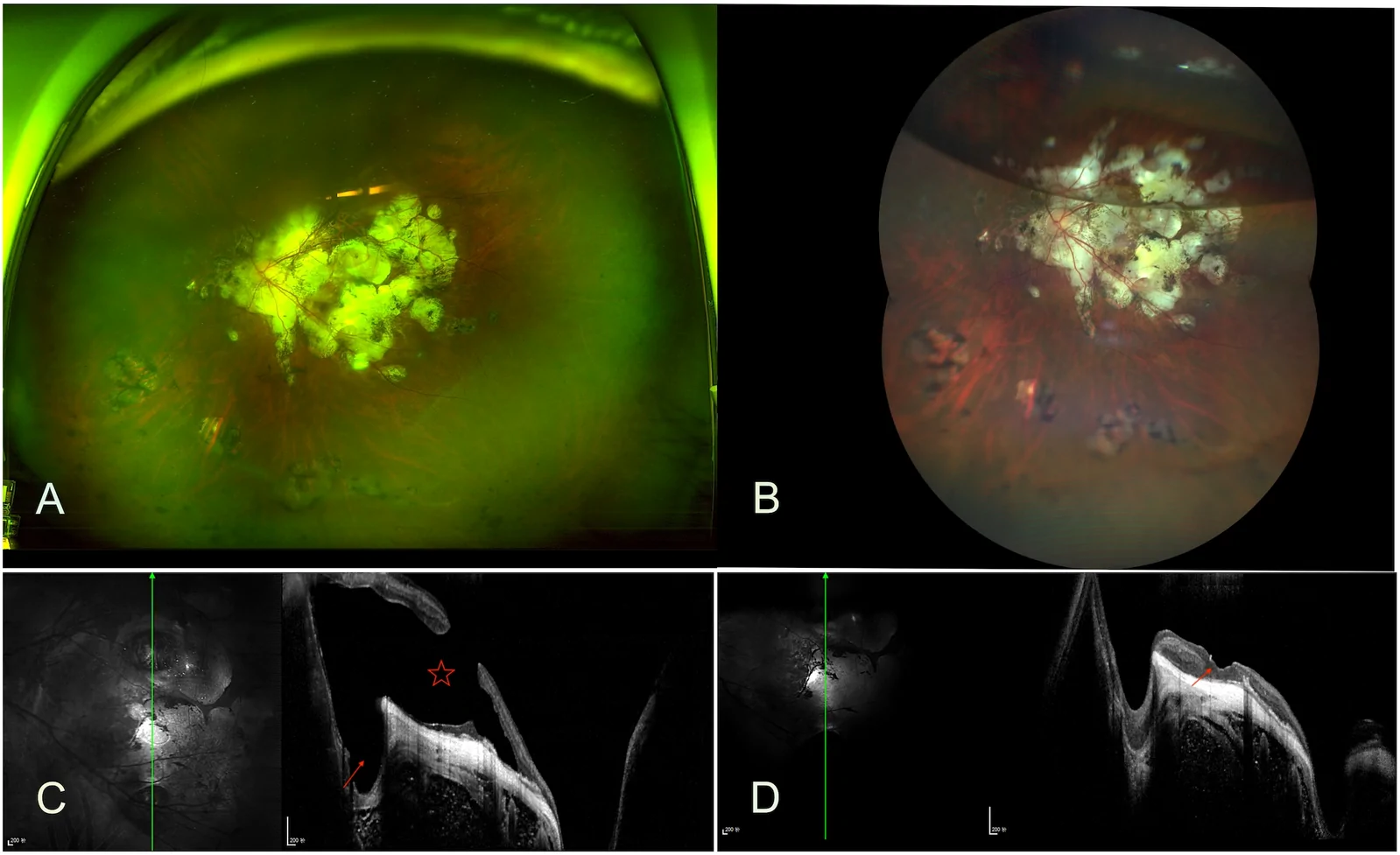
**(A)** Preoperative color fundus photograph of the right eye demonstrating a full-thickness macular hole with localized retinal detachment, associated with both superior and inferior posterior scleral staphylomas. **(B)** Fundus photograph obtained 2 weeks after surgery demonstrating complete retinal reattachment and successful closure of the macular hole. **(C)** Preoperative spectral-domain optical coherence tomography (SD-OCT) image revealing a large full-thickness macular hole (minimum diameter, 687 μm) with associated epiretinal proliferation and focal retinal detachment (red star) secondary to a superior posterior scleral staphyloma (red arrow). **(D)** SD-OCT image confirming anatomical closure of the macular hole, with the foveal defect covered by epiretinal proliferation tissue (red arrow), at 2 weeks postoperatively.

Postoperatively, the patient maintained a face-down position for 2 weeks. At the 2-week follow-up, BCVA improved from hand motions to 20/200. Fundus examination demonstrated complete retinal reattachment with successful closure of the macular hole (**Figure 1B**). SD-OCT confirmed anatomical closure of the macular hole, with the inverted ERP flap covering the foveal defect (**Figure 1D**). At the 12-month final follow-up, BCVA remained stable at 20/200. SD-OCT demonstrated persistent anatomical closure of the macular hole, with the epiretinal proliferation flap continuously covering the foveal defect. The inner retinal layers remained slightly thickened, and the ellipsoid zone showed partial but incomplete restoration (**Figure 2**). No macular hole reopening or recurrent retinal detachment was observed.

**Figure 2:**
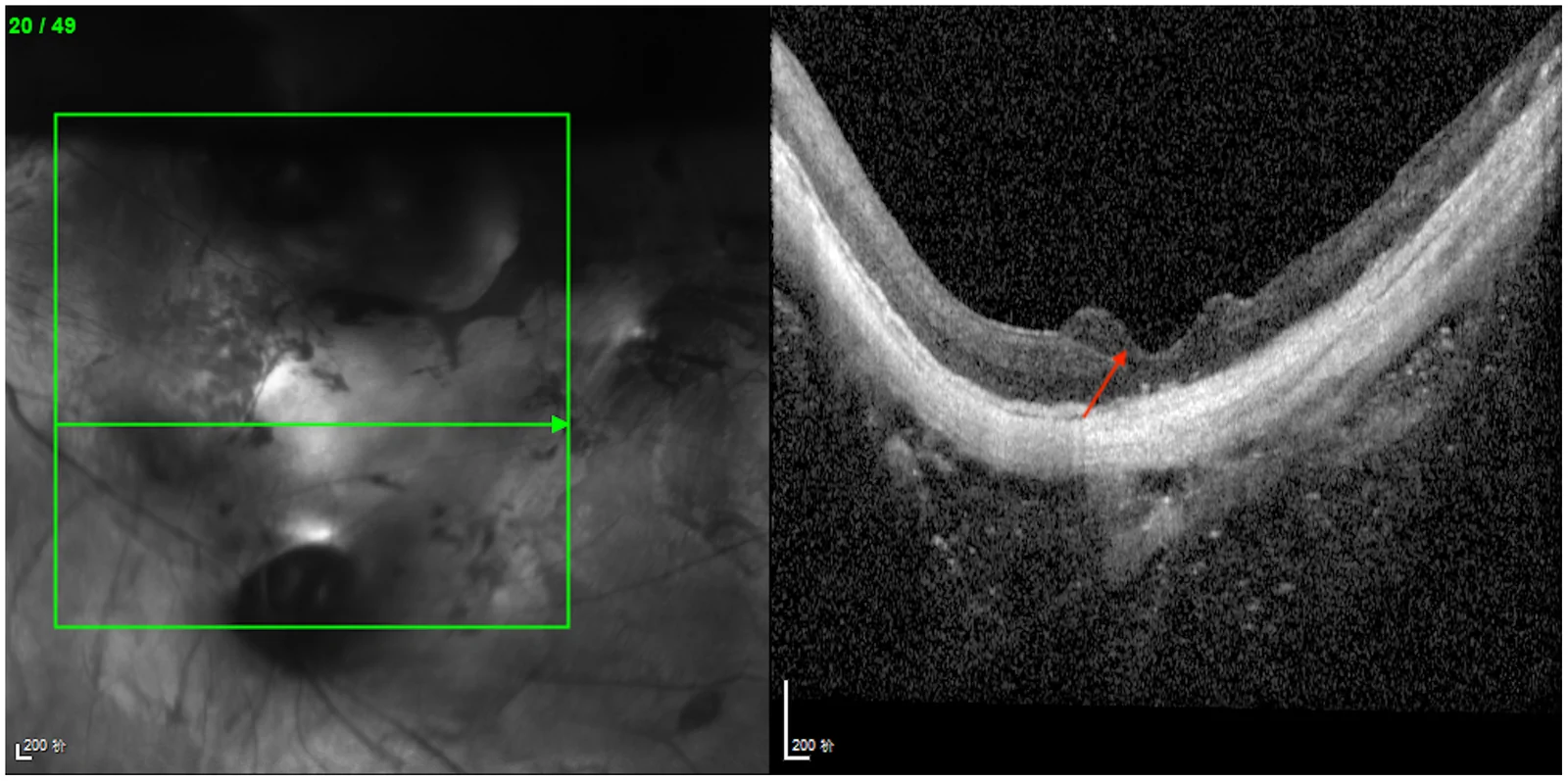
SD-OCT image confirming anatomical closure of the macular hole, with the foveal defect covered by epiretinal proliferation tissue (red arrow), at 12 months postoperatively.

## Discussion

MHRD remains one of the most challenging complications of high myopia due to progressive axial elongation, posterior scleral staphyloma, and retinal thinning, which create a biomechanical environment unfavorable for macular hole closure and retinal reattachment (10). Although pars plana vitrectomy with internal limiting membrane (ILM) peeling effectively relieves tangential traction, anatomical and functional outcomes remain less favorable than those of idiopathic macular holes, particularly in eyes with advanced myopic degeneration. The inverted ILM flap technique has substantially improved closure and retinal reattachment rates and is now regarded as the preferred approach for large or complex myopic macular holes (11). More recent modifications, including multilayer flaps (12), autologous blood stabilization (13), and perfluorocarbon-assisted techniques (14), further emphasize that successful repair depends not only on traction release but also on preservation of a stable biological scaffold.

While these techniques expand surgical options for refractory cases, they require additional donor tissue or exogenous materials and are associated with greater technical complexity. In contrast, ERP represents an intrinsic tissue already present at the pathological site. Unlike conventional epiretinal membrane, ERP is closely adherent to the retinal surface and can be mobilized as a pedicled flap without additional tissue harvesting. Müller cell-derived processes and transdifferentiated Müller cells contribute to epiretinal membranes and may serve as a scaffold substrate during retinal remodeling (15). In response to retinal injury, reactive Müller cell gliosis may be neuroprotective by stabilizing retinal architecture and promoting wound repair, but excessive activation may also contribute to glial scar formation, abnormal retinal remodeling, and persistent structural alterations. Therefore, preserving ERP should not be considered universally beneficial; rather, its potential advantages likely depend on controlled remodeling without excessive gliosis. In our patient, no recurrent retinal detachment, excessive membrane contraction, or other ERP-related complications were observed during the one-year follow-up. Larger studies are required to determine the long-term safety of this approach in the future.

From our experience, a usable ERP flap should possess sufficient thickness and fibrocellular density to withstand gentle forceps manipulation without tearing, and a clearly discernible margin that facilitates precise circumferential dissection. Beyond inner retinal reconstruction, the pathophysiology of myopic MHRD is largely driven by progressive posterior scleral ectasia, which imposes chronic mechanical stress on the outer retina and retinal pigment epithelium (RPE). This biomechanical mismatch contributes to photoreceptor stretching, disruption of the ellipsoid zone, and impaired RPE-photoreceptor interactions, all of which are important determinants of postoperative visual recovery. Consequently, successful anatomical closure does not necessarily indicate complete restoration of outer retinal integrity. In our case, retinal reattachment and macular hole closure were achieved; however, the postoperative visual improvement remained limited to 20/200, which may reflect persistent outer retinal and EZ alterations despite successful reconstruction of the foveal architecture. Conversely, individuals with scant or markedly atrophic ERP are poor candidates; in such cases, conventional inverted ILM flap or alternative graft techniques remain more appropriate.

Our patient presented a particularly complex anatomical configuration, with high myopia, a large macular hole, localized retinal detachment, and coexisting superior and inferior posterior scleral staphylomas. Instead of creating an ILM flap, we preserved the ILM and inverted the surrounding ERP to cover the macular hole. This approach avoided additional manipulation of the markedly thinned myopic retina while utilizing native proliferative tissue as the reconstructive scaffold. In lamellar macular hole surgery, ERP embedding without ILM peeling has been advocated to facilitate foveal reconstruction (16). Although the contribution of ILM preservation cannot be determined from a single case, maintaining the residual ILM may preserve the inner retinal framework while reducing iatrogenic trauma (16). This approach is unlikely to replace the inverted ILM flap technique but may be particularly suitable for eyes with abundant ERP and severely thinned retina in which additional ILM manipulation is undesirable.

This report has several limitations, including its single-case design and relatively short-term follow-up. Furthermore, the relative contributions of ERP inversion and ILM preservation cannot be distinguished. Nevertheless, this case demonstrates the feasibility of using native ERP as an autologous scaffold in a highly selected patient with MHRD and high myopia. Prospective studies involving larger cohorts are warranted to determine the reproducibility, optimal indications, and long-term anatomical and functional outcomes of this tissue-preserving strategy.

## Conclusion

This case suggests that an inverted ERP flap with preservation of the ILM may serve as a feasible tissue-preserving surgical option for selected patients with myopic MHRD. Further studies are required to clarify patient selection criteria and long-term outcomes.

## Data Availability

The raw data supporting the conclusions of this article will be made available by the authors, without undue reservation.

## Supplementary material

Supplementary Video 1 Intraoperative demonstration of 27-gauge pars plana vitrectomy using an inverted epiretinal proliferation flap technique with deliberate preservation of the internal limiting membrane for repair of a macular hole–associated retinal detachment in an eye with high myopia.
